# Illinois State Medical Society—Annual Meeting

**Published:** 1873-08

**Authors:** 


### Reports of Societies.

### *Illinois State Medical Society—Annual Meeting.*

The twenty-third annual meeting of the Illinois State Medical Society, convened in Bloomington, May 20, 1873.

The delegates and members assembled in Durley Hall at 10½ o'clock A.M. The President, Dr. D. W. Young, of Aurora, occupying the chair, called the meeting to order, and introduced Rev. Dr. Wilson, who offered prayer.

Dr. T. F. Worrell, Chairman of the Committee of Arrangements, was then introduced, and delivered a brief address of welcome on behalf of the citizens of Bloomington. This was responded to, on behalf of the Association, by Prof. E. Andrews, of Chicago.

Brief addresses of welcome were also offered by Ira J. Bloomfield, on behalf of the municipal authorities; on behalf of the Board of Education, by J. A. Jackman, Esq., which were responded to by members of the Society, after which the meeting adjourned until 2 o'clock P. M.

On reassembling at 2 o'clock the Society proceeded to business. The Committee on Credentials made their report, and the roll of officers of committees was called by the Secretary.

The first paper read was the report on Surgery, by J. L. White, M.D., of Bloomington. This was an able and interesting report, and elicited considerable discussion.

Dr. Hamilton next presented a report on Fractures, supplementary to the report on Surgery. It was an extended paper, mainly made up of statistics.

Dr. E. L. Holmes read the report of the Committee on Ophthalmology. The Secretary also read an interesting paper on the Uses of Strychnia, prepared by Dr. F. C. Holtz, of Chicago.

The Society then adjourned until 7½ o'clock P. M., when they reassembled to listen to the address of Dr. Geo. T. Allen, on the "Civilization of the Age."

Following this address, Dr. McFarland read a paper devoted mainly to the subject of the Jurisprudence of Insanity.

The Society reassembled again at 8½ o'clock A. M. The first business in order was the report of the Treasurer, Dr. Hollister.

The report of Dr. Hamilton, on Fractures, was again called up and discussed at length by various members, particularly the points in relation to the possibility of preventing shortening in oblique fractures of the femur. It was the pretty unanimous opinion of the members that some degree of shortening would almost invariably and unavoidably result, although the statistics given in the report of Dr. Hamilton seemed to favor a contrary opinion.

The report on Drugs and Medicines, by Dr. Hollister, was next read and discussed. Dr. Prince, of Jacksonville, also submitted a report on Galvano-Therapeutics.

The Committee on Nominations submitted the following report:

For President, Dr. T. F. Worrell, of Bloomington; 1st Vice-President, Dr. E. L. Holmes; 2d Vice-President, Dr. Birney; Treasurer, Dr. J. H. Hollister. Next place of meeting, Chicago.

*Committee on Practical Medicine*—Dr. H. Crawford, Monmouth; Dr. John Wright, DeWitt; Dr. Van Horn, Jerseyville.

*Surgery*—Drs. W. P. Pierce, P. Raskatin, E. R. Willard.

*Obstetrics*—Drs. S. E. Plummer, C. T. Hassian, Robert Beal.

*Drugs and Medicine*—Drs. W. E. Quine, Chicago; R. W. James, Danville; E. P. Cook, Mendota.

*Ophthalmology*—Drs. E. L. Holmes, J. P. Johnson, F. C. Holtz.

*Otology*—Drs. S. J. Jones, Chas. Hunt, Thos. Galt.

*Special Committee on Idiocy*—Dr. C. F. Wilber.

*Galvano-Therapeutics*—Dr. D. Prince.

*Pathology of Nervous System*—Dr. C. W. Earle.

*Dermatology*—Dr. Chas. G. Smith.

*Phthisis Pulmonalis*—Dr. J. H. Hollister.

*Strictures of Urethra*—Prof. Andrews.

*Diseases of Respiratory Organs*—Dr. Daniel Lichty.

*Committee of Arrangements*—Drs. N. S. Davis, W. E. Quine, Chas. G. Smith, E. Powell.

*Assistant Secretary*—Dr. W. E. Quine.

The report of the Nominating Committee was unanimously adopted.

A report on "The Assistance Necessary and Justifiable in Difficult and Protracted Labor," by Dr. S. D. Hawley, was read by the Secretary. The report elicited extended and various criticism and discussion, which space does not permit us to report. The Society again adjourned until evening, the afternoon being spent, at the invitation of the Committee of Arrangements, in visiting the State Normal School, the Soldiers' Orphans' Home, and the C. A. & St. L. R. R. machine shops. At each of these places the Society was welcomed by the authorities in charge.

The Association reassembled in the evening session at 8 o'clock P. M.

Dr. Worrell read a report upon Hygiene, and in connection with it a supplementary report, prepared by Dr. N. S. Davis, on the Hygienic Management of Children.

A report on the Physiology of the Nervous System, by Dr. Earle, the report on Otology, by Dr. S. J. Jones, and a report on Obstetrics, from Dr. T. D. Fitch, completed the list of reports presented.

On motion of Dr. Hollister, the Association proceeded to the election of delegates to the American Medical Association, the following named being chosen:

Drs. A. H. Luce, J. O. Hamilton, E. Goodbrake, E. P. Cook, W. P. Pierce, D. Prince, C. T. Homer, J. H. Hollister, W. W. McMann, S. H. Birney, C. W. Earle, J. B. Walker, Haller, W. E. Quine, E. R. Willard, Whitmeyer, E. R. Travers, J. P. Mathews, E. W. Hewett, D. Young, T. D. Fitch, O. W. Moon, John Lyttle, A. C. Rankin, O. Everett, F. H. Davis, J. G. Curtis, S. E. Plummer, F. M. Wilder, J. L. White, A. S. McArthur, N. B. Cole, D. McVey, C. Armstrong, and A. McFarland.

Dr. Fitch moved that the Association proceed to the election of delegates to State Medical Societies. The motion was carried, and the following persons were elected:

Drs. G. W. Jones and Birney, to Indiana Medical Society; Drs. Gray and Ellsbury, to Ohio; Drs. M. Gunn and E. L. Holmes, to Michigan; Drs. McArthur and Irwin, to Wisconsin; Drs. T. D. Fitch and J. Galt, to Iowa; Drs. C. R. Cook and J. O. Hamilton, to Missouri; Drs. T. F. Worrell and E. R. Willard, to Minnesota.

A motion from Dr. Hollister that the annual assessment be fixed at $3 for the year, was carried.

After passing resolutions of thanks to the Committee of Arrangements, the municipal authorities, and others who had assisted in entertaining the Association, the meeting was adjourned *sine die.*—*Med. Examiner.*

